# The steroid-sparing effect of JAK inhibitors across multiple patient populations

**DOI:** 10.3389/fimmu.2024.1376476

**Published:** 2024-04-12

**Authors:** Paola Conigliaro, Clara Minerba, Andrea Vendola, Luigi Fiannacca, Paola Triggianese, Barbara Kroegler, Elisabetta Greco, Alberto Bergamini, Maria Sole Chimenti

**Affiliations:** U.O.C. Reumatologia, Dipartimento di Medicina dei Sistemi, Universitá di Roma “Tor Vergata”, Roma, Italy

**Keywords:** rheumatoid arthritis, psoriatic arthritis, JAK inhibitors, glucocorticoids, retention rate, prognostic factors

## Abstract

**Introduction:**

JAK-inhibitors (JAK-i) represent an effective treatment in Rheumatoid Arthritis (RA) and Psoriatic Arthritis (PsA). Oral glucocorticoids (OGC) are commonly used in combination with JAK-i to reach therapeutic target. We aimed to assess, in a real-life setting, the reduction of OGC dose during JAK-i treatment in active RA and PsA patients.

**Methods:**

We prospectively enrolled 103 patients (88 RA, 15 PsA) treated with JAK-i: 24% bio-naïve (b-naïve), 76% bDMARD-insufficient responders (bDMARD-IR) and 40% difficult to treat (D2T), defined as failure of ≥2 bDMARDs with different mechanism of action. Disease activity (DAS28 and DAPSA, VAS-pain, GH) and OGC dose was collected at baseline and after 3, 6 and 12 months (T3, T6, T12) of treatment.

**Results:**

In all the cohort and in b-naïve patients we reported a reduction of OGC dose at all time-points; bDMARD-IR patients were able to reduce OGC dose at T3 and T12; D2T ones only at T3. We reported an improvement of disease activity and withdrawal of OGC as early as three months of therapy, at all time-points, regardless of line of bDMARD treatment.

**Conclusion:**

Chronic OGC may cause detrimental bone, metabolic, cardiovascular side effects and infections; therefore JAK-i steroid-sparing effect may be beneficial for patients in long-term treatment.

## 1 Introduction

In recent years, there has been an important expansion of the therapeutic armamentarium for rheumatoid arthritis (RA) and psoriatic arthritis (PsA). This wide range of treatment options allows clinicians to select optimal therapies for patients, with the aims of achieving remission or low disease activity (LDA) and preventing joint damage. Among emerging therapies, Janus kinase inhibitors (JAK-i) are a class of oral targeted disease-modifying drugs (tsDMARDs) that are promising in treating inflammatory arthritis (1). JAK-i are small molecules that inhibit the intracellular signaling pathway JAK–STAT, crucially involved in the inflammatory cascade and immune response. So far, four JAK-i (baricitinib, tofacitinib, upadacitinib, filgotinib) are approved for the treatment of RA, three (tofacitinib, upadacitinib, and filgotinib) for PsA, while other JAK-i are under investigation. Current therapeutic guidelines place JAK-i at the same level as biological antirheumatic drugs (bDMARDs), after the failure of conventional disease-modifying drugs (csDMARDs) for RA patients (2) and after the failure of at least one bDMARD, as a second-line treatment, for PsA patients (3). JAK-i efficacy in the treatment of moderate–severe RA and PsA has been assessed in randomized clinical trials (4–8). The most common side effect observed during JAK-i treatment has been infections, especially of the upper respiratory tract. In addition, reactivation of the herpes zoster virus (HZV) and alteration in the blood lipid profile have typically been reported under JAK-i therapy (9, 10). The Pharmacovigilance Risk Assessment Committee (PRAC) of the European Medicines Agency (EMA) has recently provided updated recommendations for the use of all JAK-i approved in Europe, with a warning for patients older than 65 years old, smokers, and patients with increased risk of major cardiovascular events, cancer, and blood clots in the lungs and deep veins (11, 12). Oral glucocorticoids (OGCs) are indicated in the acute management of RA flares; even though their chronic use is discouraged for their cumulative metabolic, cardiovascular, bone, and infective detrimental effects, in clinical practice, OGCs are commonly used for long-lasting therapies. CsDMARDs are often associated with bDMARDs and tsDMARDs, and combination treatments are characterized by a lower target specificity and present risk of infections and gastrointestinal and hematological side effects (13). In this real-life study, we aim to investigate the potential JAK-i steroid-sparing effect across different multiple patient populations.

## 2 Materials and methods

Consecutive patients with active RA or PsA treated with first JAK-i were prospectively enrolled in a single Italian rheumatology center (Policlinico di Roma “Tor Vergata”, Rome, Italy), from 1 September 2021 to 30 June 2022. Eligible patients had to 1) be ≥18 years old, 2) fulfill the 2010 ACR/EULAR classification criteria (14) for RA or the CASPAR criteria (15) for PsA, and 3) have had an insufficient previous response or intolerance to at least one csDMARD. Patients with any missing data were excluded from the analysis. The study population was first divided into three subgroups: patients who have never received any bDMARDs or tsDMARDs (b-naive), patients who failed at least one bDMARD (bDMARD-IR), and difficult-to-treat patients (D2T) including both patients who satisfy the EULAR definition of difficult-to-treat RA (16) and PsA patients which failed at least two bDMARDs with different mechanisms of action. In a second analysis, the general population was divided into patients assuming JAK-i monotherapy and patients treated with concomitant methotrexate. For all the cohorts, demographic data were registered; for RA patients, we reported the presence of RF and/or ACPA antibodies or if they were seronegative. All data were collected at baseline and after 3, 6, and 12 months (T3, T6, T12) of treatment. We reported the number of tender and swollen joints, and C-reactive protein (CRP) serum level was registered at every time point. We collected data about disease activity: Disease Activity Score in 28 joints with CRP (DAS28-CRP) (17) or Disease Activity in Psoriatic Arthritis with CRP (DAPSA-CRP) (18). DAS28-CRP/DAPSA-CRP scores were used to assess remission (<2.6 for DAS28, ≤4 for DAPSA): low disease activity (LDA: ≥2.6 and ≤3.2 for DAS28, >4 and ≤14 for DAPSA), moderate disease activity (MDA: >3.2 and ≤5.1 for DAS28, >14 and ≤28 for DAPSA), and high disease activity (>5.1 for DAS28, >28 for DAPSA) according to the EULAR/ACR recommendations (19, 20). For the statistical analysis of disease activity, the PsA population was not divided into subgroups because of the small size of the cohort. All patients were questioned at baseline and at each time point about articular pain by using a Visual Analog Scale (VAS-pain) (21) considering “no pain at all” as score 0 and “worst imaginable pain” as score 100 and about global health by using the General Health score (GH) (22) considering “best health status” as score 0 and “worst health status” as score 100. Radiographs of the hands and feet were obtained at the time of inclusion, and they were evaluated by an experienced reader for the presence or absence of erosions. Concomitant oral glucocorticoid daily dose was also registered at every visit, expressed in milligrams (mg) as prednisone-equivalent dose (PDN-dose). Temporary and permanent JAK-i withdrawals were registered along with the reasons why each patient suspended the treatment duration.

Written informed consent was obtained from all the patients enrolled in the study, which was conducted in accordance with the ethical principles of the Declaration of Helsinki, and the study was approved by the Scientific Ethics Committee of the University of Rome Tor Vergata, Rome, Italy.

### 2.1 Statistical analysis

Different subgroups at baseline and different time points were compared using the non-parametric Wilcoxon signed-rank test and Mann–Whitney test with paired data, respectively. A multivariable logistic regression analysis was used to correct the *p*-value for gender, age, disease duration, smoking habit, comorbidities, and being a naive patient (padj). Median survival time and survival curves were obtained using the Kaplan–Meier curve, along with a log-rank test between the curves. *p*-values <0.05 were considered statistically significant. Statistical analysis was performed using GraphPad Prism version 9 (GraphPad Software, Inc., San Diego, CA, USA) and SPSS software version 24 for Windows (SPSS Inc., Chicago, IL, USA).

## 3 Results

Our cohort included 103 patients with active arthritis (88 RA, 15 PsA) treated with JAK-i: 45 patients received tofacitinib, 44 baricitinib, 8 upadacitinib, and 6 filgotinib. Demographic and clinimetric baseline characteristics of the study population are described in **Table 1**. The majority of the patients were women (76%) with a mean age of 60 years and an average disease duration of 11 years. Fifty-seven percent of RA cases were positive for rheumatoid factor and 48% for anti-citrullinated protein antibodies. In 56 cases, radiographic erosions of the hands and feet were present at baseline (60.2% in RA patients and 20% in PsA patients). A total of 25 (24%) patients were b-naive, 78 (76%) bDMARD-IR, and 41 (40%) difficult to treat (D2T). At baseline, 54 patients (52%) were concomitantly treated with OGC (12 b-naive, 42 bDMARD-IR, 35 D2T) with an average daily dose of 4.3 mg; 60 patients (58%) were concomitantly treated with a csDMARD.

**Table 1:** Baseline characteristics of rheumatoid arthritis and psoriatic arthritis patients receiving JAK inhibitors.

	General population (*n* = 103)	Rheumatoid arthritis (*n* = 88)	Psoriatic arthritis (*n* = 15)
**Women, *n* (%)**	78 (76%)	68 (72.3%)	10 (66.7%)
**Age (years)**	60 ± 12	60.0 ± 12.4	55.0 ± 12.4
**Disease duration (months)**	134 ± 88	129.2 ± 90.2	160.3 ± 82.8
**DAS28-CRP**	NA	4.9 ± 1.0	NA
**DAPSA-CRP**	NA	NA	41.0 ± 22.2
**VAS-pain (0–100)**	73.1 ± 14.5	69.6 ± 15.9	66.1 ± 14.6
**GH (0–100)**	69.4 ± 14.2	68.4 ± 15.6	66.1 ± 13.6
**Tender joints (*n*)**	10.6 ± 6.4	10.1 ± 6.3	13.3 ± 5.5
**Swollen joints (*n*)**	4.1 ± 5.4	4.4 ± 5.5	3.0 ± 3.6
**Erosions, *n* (%)**	56 (54.3%)	53 (60.2%)	3 (20%)
**CRP (mg/dl)**	0.9 ± 1.2	1.0 ± 1.2	0.6 ± 0.8
**JAK-i monotherapy, *n* (%)**	43 (42%)	33 (37.5%)	10 (66.7%)
**PDN equivalent dose per day (mg)**	4.3 ± 5.4	4.6 ± 5.5	5.2 ± 4.7

Categorical variables are expressed as number (%); continuous variables are expressed as mean ± standard deviation.

DAS28-CRP, Disease Activity Score in 28 joints with CRP; NA, not applicable; DAPSA-CRP, Disease Activity in Psoriatic Arthritis with CRP; VAS-pain, Visual Analogic Scale; GH, patient General Health score.

**Table 2** shows the baseline characteristics of the enrolled population considered as a whole and divided into the following subgroups: b-naive, bDMARD-IR, and D2T. At baseline, RA patients presented moderate disease activity, as shown by DAS28-CRP in the RA cohort and all subgroups, whereas PsA patients presented high disease activity, according to DAPSA-CRP in all categories. At baseline, the CRP serum level of b-naive patients was higher than the other subgroups, while the patients’ GH, VAS-pain, and DAS28-CRP were significantly higher in D2T patients than in b-naive patients (*p* = 0.0029, *p* = 0.0020, and *p* = 0.02, respectively).

**Table 2:** Baseline clinimetrics of rheumatoid arthritis and psoriatic arthritis patients receiving JAK inhibitors in the study population and in the three subgroups: bio-naive (b-naive), bDMARD-insufficient responders (bDMARD-IR), and difficult to treat (D2T).

	General population (*n* = 103)	b-naive (*n* = 25)	bDMARD-IR (*n* = 78)	D2T (*n* = 41)
**Tender joints (*n*)**	10.6 ± 6.4	10.8 ± 6.3	10.6 ± 6.4	11.9 ± 6.4
**Swollen joints (*n*)**	4.1 ± 5.4	3.7 ± 5.4	4.3 ± 5.4	5.1 ± 5.4
**CRP (mg/dl)**	0.9 ± 1.2	1.4 ± 1.2	0.8 ± 1.2	0.7 ± 1.2
**GH (0–100)**	67.6 ± 14.2	62.5 ± 14.0	69.2 ± 14.1	69.4 ± 14.2
**VAS-pain (0–100)**	68.8 ± 14.5	60.2 ± 14.5	71.5 ± 14.5	73.1 ± 14.5
**DAS28-CRP**	4.7 ± 1	4.7 ± 1.0	4.7 ± 0.9	4.9 ± 1.1
**DAPSA-CRP**	39.6 ± 19.3	42.3 ± 6.8	39.2 ± 19.3	40.1 ± 19.3

Categorical variables are expressed as number (%); continuous variables are expressed as mean ± standard deviation.

CRP, C-reactive protein; DAS28-CRP, Disease Activity Score in 28 joints with CRP; DAPSA-CRP, Disease Activity in Psoriatic Arthritis with CRP; VAS-pain, Visual Analog Scale; GH, patient General Health score.

We observed a significant improvement of disease activity by VAS-pain and GH at all time points in the whole population (*p* < 0.001, *p* < 0.05, *p* < 0.0001, *p* < 0.001, respectively, **Table 3**). When patients were divided according to the diagnosis, we observed that RA patients showed a reduction of DAS28-CRP, VAS-pain, and GH at all time points (*p* < 0.0001 for all comparisons), while PsA patients exhibited a reduction of DAPSA-CRP at all time points (*p* < 0.05 for all comparisons) and of VAS-pain at T3 (*p* = 0.01) and T6 (*p* = 0.04) (**Supplementary Table 1**).

**Table 3:** Disease activity of rheumatoid arthritis and psoriatic arthritis patients receiving JAK inhibitors during the follow-up in the study population (a) and in the three subgroups: (b) bio-naive (b-naive), (c) bDMARD-insufficient responders (bDMARD-IR), and (d) difficult to treat (D2T).

a)						
**General population (n = 103)**						
	**Baseline (n = 103)**	**T3 (n = 103)**	**T6 (n = 84)**		**T12 (n = 60)**	
**DAS28-CRP**	4.87 ± 1	3.7 ± 1.1***	3.4 ± 0.9***		3.2 ± 0.9***	
**DAPSA-CRP**	41.0 ± 22.2	37.6 ± 22.2*	32.7 ± 15.1*		27.24 ± 3.5*	
**VAS-pain**	73.1 ± 14.5	50.7 ± 19.4***	43.4 ± 19.9***		36.5 ± 17.3***	
**GH**	69.4 ± 14.2	49.0 ± 17.6***	45.3 ± 20.3***		41.3 ± 15.3***	
b)						
**b-naive (n = 25)**						
	**Baseline (n = 25)**	**T3 (n = 25)**	**T6 (n = 22)**		**T12 (n = 16)**	
**DAS28-CRP**	4.75 ± 1	2.93 ± 1.1***	2.85 ± 0.9***		2.87 ± 0.9**	
**VAS-pain**	60.6 ± 14.6	45.9 ± 19.5***	31.2 ± 20.0***		27.1 ± 15.7***	
**GH**	62.5 ± 14.0	38.6 ± 17.9***	32.3 ± 20.3***		32.8 ± 14.2***	
c)						
**bDMARD-IR (n = 78)**						
	**Baseline (n = 78)**	**T3 (n = 78)**	**T6 (n = 62)**		**T12 (n = 44)**	
**DAS28-CRP**	4.7 ± 1	3.6 ± 1.1***	3.2 ± 0.9***		3.0 ± 0.9***	
**VAS-pain**	71.5 ± 14.5	47.9 ± 19.3***	40.3 ± 19.9***		36.9 ± 17.5***	
**GH**	69.2 ± 14.2	48.9 ± 17.6***	43.4 ± 20.3***		38.9 ± 15.8***	
d)						
**D2T (n = 41)**						
	**Baseline (n = 41)**	**T3 (n = 41)**	**T6 (n = 34)**		**T12 (n = 24)**	
**DAS28-CRP**	4.9 ± 1.0	3.7 ± 1.1***	3.4 ± 0.9***		3.2 ± 0.9***	
**VAS-pain**	73.1 ± 14.5	50.7 ± 19.4***	43.4 ± 19.9***		36.5 ± 17.3***	
**GH**	69.4 ± 14.2	49.0 ± 17.7***	45.3 ± 20.3***		41.3 ± 15.6***	

Continuous variables are expressed as mean ± standard deviation. Comparisons were performed with baseline values, *p < 0.05, **p < 0.01, ***p < 0.001 vs. baseline (Wilcoxon signed-rank test).

DAS28-CRP, Disease Activity Score in 28 joints with CRP; DAPSA-CRP, Disease Activity in Psoriatic Arthritis with CRP; VAS-pain, Visual Analog Scale; GH, patient General Health score; T3, 3 months; T6, 6 months; T12, 12 months.

A significant reduction of DAS28-CRP, VAS-pain, and GH was observed in b-naive (*p* < 0.002, *p* < 0.001, and *p* < 0.0001, respectively), bDMARD-IR (*p* < 0.0001 for all the measures at all time points), and D2T patients (*p* < 0.0006 for DAS28-CRP, *p* < 0.0001 for the others). We did not array patients in the three subgroups for the DAPSA-CRP score because of the small number of PsA patients (**Table 3**). Remission was achieved in the whole cohort in 19.5% of patients (16/82) at T3, 31% (20/65) at T6, and 30% (15/50) at T12. Likewise, LDA was gained in 36.5% (30/82) at T3, 54% (35/65) at T6, and 66% (33/50) at T12.

In the multivariable analysis (**Table 4**), being b-naive patients was an independent factor for achieving remission at T3 and T6 (T3: padj = 0.04, OR 4.9 and T6: padj = 0.04, OR 5.3). Likewise, the presence of comorbidities was a negative predicting factor of achieving LDA at T3 (padj = 0.01, OR 0.4) and both remission and LDA at T6 (rem: padj = 0.04, OR 0.5 and LDA: padj = 0.002, OR 0.3). Moreover, female sex was a negative prognostic factor to gain LDA at T6 (padj = 0.02, OR 0.1) and remission at T12 (padj = 0.04, OR 0.2).

**Table 4:** Factors associated with remission and low disease activity in the follow-up.

	Remission T3			LDA T3			Remission T6			LDA T6			Remission T12			LDA T12		
	*p*	OR	CI	*p*	OR	CI	*p*	OR	CI	*p*	OR	CI	*P*	OR	CI	*p*	OR	CI
**Female**	0.6	0.7	0.1–3.1	0.9	0.9	0.2–3.5	0.3	0.5	0.1–2.2	**0.02**	0.1	0.02–0.7	**0.04**	0.2	0.02–1	0.3	0.4	0.06–2.6
**Age**	0.9	0.9	0.9–1	0.3	0.9	0.9–1	0.5	1	0.9–1.1	0.4	1	0.9–1.1	0.8	0.9	0.9–1.1	0.4	1	0.9–1.1
**Disease duration**	0.6	1	0.9–1	0.4	1	0.9–1	0.3	1	0.9–1	0.3	1	0.9–1	0.5	0.9	0.9–1	0.7	0.9	0.9–1
**Smoking history**	0.8	1.2	0.3–4.7	0.1	0.3	0.05–1.4	0.9	1.1	0.2–5.3	0.1	0.2	0.03–1.5	0.5	0.5	0.07–3.6	0.08	0.2	0.03–1.2
**Comorbidities**	0.4	0.5	0.07–2.9	**0.01**	0.4	0.003–0.5	**0.04**	0.5	0.3–1	**0.002**	0.3	0.2–0.7	0.1	0.6	0.3–1.2	0.4	0.8	0.4–1.3
**naïve**	**0.04**	4.9	0.9–27.6	0.6	1.3	0.4–4.8	**0.04**	5.3	0.8–34.2	**0.04**	7.6	0.9–60	0.5	2	0.3–14	0.9	1	0.1–6.8

p, p-value; OR, odds ratio; CI, confidence interval.Significant p values are indicated in bold.

**Figure 1:**
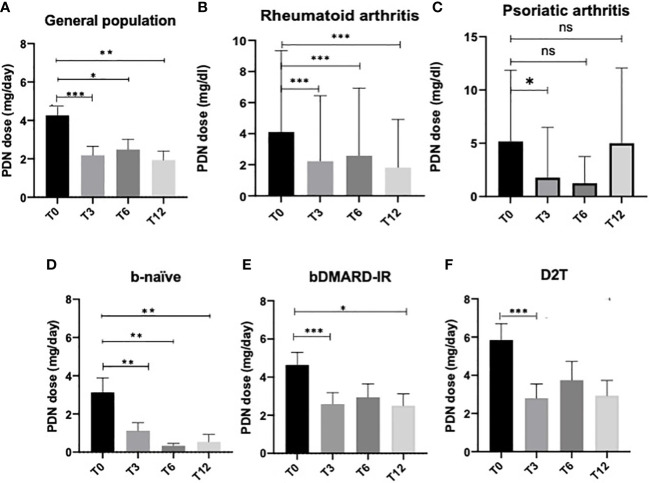
Oral glucocorticoid dose at all time points in the general population **(A)**, rheumatoid arthritis patients **(B)**, psoriatic arthritis patients **(C)** receiving JAK inhibitors, and in the three subgroups: **(D)** bio-naive (b-naive), **(E)** bDMARD-insufficient responders (bDMARD-IR), and **(F)** difficult to treat (D2T). Oral glucocorticoid daily dose is expressed as the mean prednisone-equivalent dose in milligrams (PDN dose) ± standard error. T3, 3 months; T6, 6 months; T12, 12 months. *p < 0.05, **p < 0.01, ***p < 0.001 vs. baseline (Wilcoxon signed-rank test).

In all the cohorts, we reported a statistically significant reduction of PDN dose at T3 (*p* < 0.0001), T6 (*p* = 0.02), and T12 (*p* = 0.002, **Figure 1A**). When patients were divided according to the diagnosis, RA patients reduced the steroid dose at all time points of the follow-up (T0 vs. T3: *p* < 0.0001, T0 vs. T6: *p* = 0.007, T0 vs. T12: *p* = 0.0007; **Figure 1B** and **Supplementary Table 2**), while PsA patients reduced the steroid dose at 3 months of follow-up (T0 vs. T3: *p* = 0.03, **Figure 1C** and **Supplementary Table 2**). Likewise, b-naive patients were able to reduce PDN dose at all time points (*p* < 0.01 for all comparisons, **Figure 1D**). bDMARD-IR patients were able to significantly reduce PDN dose at T3 (*p* < 0.0001) and T12 (*p* = 0.03, **Figure 1E**); the reduction of OGC dose in D2T patients was statistically significant only at T3 (*p* = 0.0001, **Figure 1F**).

In patients who completed the follow-up, withdrawal of OGC was observed at all time points in each subgroup. The steroid withdrawal rate was higher for all subgroups in the first 3 months of therapy (**Table 5**).

**Table 5:** Oral glucocorticoid dose and withdrawal at all time points in the study population and in three subgroups: bio-naive (b-naive), bDMARD-insufficient responders (bDMARD-IR), and difficult to treat (D2T).

	OGC dose (mg/day)				OGC withdrawal (*n*/tot, %)			
	T0	T3	T6	T12	T0	T3	T6	T12
General population	4.3 ± 5.4	2.2 ± 4.2***	2.5 ± 4.3**	1.9 ± 3.3**	/	28/54 (52%)	9/26 (35%)	9/17 (53%)
b-naive	3.1 ± 5.4	1.1 ± 4.3**	1.4 ± 4.3**	0.5 ± 3.2**	/	5/12 (42%)	2/7 (29%)	5/5 (100%)
bDMARD-IR	4.6 ± 4.3	2.6 ± 4.3***	2.9 ± 3.3	2.5 ± 2.9*	/	21/42 (50%)	9/21 (43%)	4/12 (33%)
D2T	5.8 ± 5.4	2.8 ± 3.8***	3.7 ± 4.3***	2.9 ± 3.3*	/	18/35 (51%)	7/17 (41%)	4/10 (25%)

Oral glucocorticoid daily dose is expressed as the mean prednisone-equivalent dose in milligrams (PDN dose) ± standard deviation; OGC withdrawal rate is expressed as number of patients who suspended OGC/number of patients assuming oral glucocorticoid at the beginning of the period considered. Comparisons were performed with baseline values; *p < 0.05, **p < 0.01, ***p < 0.001 vs. baseline (Wilcoxon signed-rank test).

T3, 3 months; T6, 6 months; T12, 12 months.

In order to identify factors associated with steroid withdrawal, univariable analysis was performed showing a higher prevalence of non-smokers in patients who suspended steroids during 1 year of follow-up (86% vs. 14%, *p* = 0.01), and these data were confirmed in the multivariable analysis when corrected for sex, age, BMI, and disease duration (padj = 0.009, OR = 0.06, 95% CI 0.007–0.5). Steroid withdrawal did not differ according to sex, BMI, disease duration, number of comorbidities, use of JAK-i in monotherapy or in combination with csDMARD treatment, and being b-naive, bDMARD-IR, or D2T patients.

### 3.1 JAK inhibitors in monotherapy and in combination with csDMARDs

**Figure 2:**
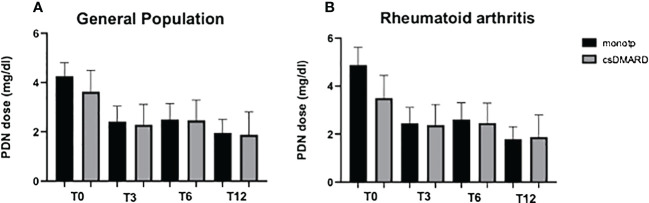
Oral glucocorticoid dose at all time points in the general population **(A)** and in RA patients **(B)** receiving JAK inhibitors; the study population was arrayed in patients with JAK-i monotherapy (monotp) and with concomitant csDMARD (w/csDMARD). Oral glucocorticoid daily dose is expressed as the mean prednisone-equivalent dose in milligrams (PDN). csDMARD, conventional synthetic disease-modifying antirheumatic drug; T3, 3 months; T6, 6 months; T12, 12 months.

A significant reduction of OGC daily dose was observed in all the cohorts regardless of the concomitant therapy with any csDMARD at all time points. In patients with JAK-i monotherapy (monotp, *n* = 43), a significant reduction of OGC dose was observed at T3, T6, and T12 (*p* < 0.0001, *p* = 0.0246, and *p* = 0021, respectively). Likewise, in patients with a concomitant csDMARD (w/csDMARD, *n* = 60), OGC dose was reduced at all time points (*p* = 0.0275, *p* = 0.0377, and *p* = 0.2717, respectively) (**Figure 2A**). OGC dose did not significantly differ at any time point between patients on monotherapy and patients w/csDMARD; only at baseline, patients on monotherapy had a higher OGC daily dose than patients w/csDMARD (*p* = 0.040). When RA patients were analyzed separately, those treated with JAK-i in monotherapy were able to taper their steroid dose throughout the entire follow-up period (T0 vs. T3: *p* = 0.01, T0 vs. T6: *p* = 0.03, T0 vs. T12: *p* = 0.01). In contrast, the steroid dose in those patients treated with JAK-i in combination with csDMARDs did not significantly decrease during the follow-up. Additionally, at baseline, patients on monotherapy had a higher steroid dose compared with those on combination therapy (*p* = 0.03) (**Figure 2B**).

### 3.2 Suspensions and drug survival

**Figure 3:**
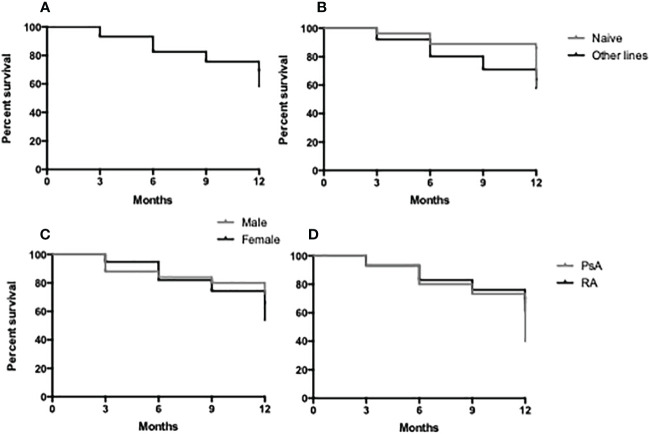
Retention rate of JAK-i therapy in 1 year of follow-up in the study population **(A)**, in bDMARD naive vs. other lines **(B)**, in male vs. female patients **(C)** and in RA vs. PsA patients **(D)**.

Crude drug survival was estimated by the Kaplan–Meier curves, and the 1-year drug survival (**Figures 3A–D**) was 79% with 21% (*n* = 22) of the patients having discontinued therapy before the end of the first year of treatment. A total of 95 patients reached 3 months of evaluation, 83 patients reached 6 months of follow-up, and 81 patients reached 1 year of therapy. The average treatment duration was 11.5 ± 10.2 months. There were no statistically significant differences in drug survival between male and female patients and according to the line of treatment (naive vs. other lines) or disease (**Figures 3B–D**).

The most common cause of discontinuation was therapeutic inefficacy, defined as both primary or secondary lack of effectiveness. In our cohort, 41% (*n* = 9) of patients suspended JAK-i therapy for inefficacy, and only one of nine patients was affected by PsA: one patient was b-naive, four bDMARD-IR, and four D2T. Patients with an insufficient therapeutic response suspended the therapy after an average of 7 ± 10.9 months, with mean inflammatory and disease activity indexes slightly higher than the whole suspension cohort (CRP 1.6 ± 5.6 mg/dl, DAS28-CRP 4.6 ± 1.2 mg/dl, DAPSA 50.1). The second most common cause of discontinuation was viral infections (*n* = 5), and there were two recurrent reactivations of herpes simplex virus (HSV). Approximately 75% (*n* = 4) of patients who suspended the therapy for viral infections were being treated with concomitant OGC, with an average PDN-equivalent daily dose higher than the whole suspension cohort (11.5 ± 6.8 mg). Intolerance to JAK-i treatment caused the discontinuation in 18% (*n* = 4) of patients, with nausea and abdominal bloating as the most common symptoms. Allergic reactions caused 14% of JAK-i suspensions (*n* = 3), and they were all mild with diffuse urticaria for two patients and lip and tongue swelling for one patient. Of particular interest, in the whole cohort, one case (4% of suspensions) of major adverse cardiovascular event (MACE) was registered: an episode of pulmonary embolism in a 61-year-old lady with hypertension after 7 months of JAK-i treatment.

## 4 Discussion

In this study, we demonstrated the clinical effectiveness and safety profile of JAK-i in Italian patients with active inflammatory arthritis with 1 year of follow-up in real-world settings. In particular, JAK-i therapy has proven effective in all examined populations, regardless of whether they were b-naive, bDMARD-IR, or D2T. This finding is particularly noteworthy, as even in our case series, patients defined as D2T exhibited worse clinimetrics at baseline compared with other patients, and in general, a substantial proportion was classified as D2T (40% of enrolled patients). Remission and LDA rates were consistent with the literature (23), with remission rates ranging from 20% to 30% and LDA rates from 36% to 66%. Multivariate analysis for identifying factors associated with achieving clinical remission demonstrated that b-naive patients achieve clinical remission five times more often than other patients (24, 25). Therefore, this observation suggests an earlier use of JAK-i right after the failure of csDMARDs. On the contrary, the presence of comorbidities and female sex were negative prognostic factors for the achievement of treatment goals. Although it is well-known that comorbidities present a considerable burden for patients with RA or PsA and that sex and gender influence inflammatory arthritis manifestations and course, further studies would be helpful to assess factors predicting the response to JAK-i therapy (26–30). Literature data suggest that some comorbidities are more prevalent in the male population, such as cardiovascular and respiratory diseases and diabetes, while others like depression and osteoporosis are more prevalent in the female population (31). In our population, the prevalence of comorbidities in general was similar between men and women. We previously demonstrated in cohorts of RA and PsA patients treated with anti-TNF that female sex and the presence of comorbidities were negative predicting factors of gaining treatment targets (32). Women with both RA and PsA have a worse disease course in terms of disease activity, loss of function, joint destruction, and work disability (33). They also appear to show a worse response to synthetic drugs and bDMARDs, although this result is debated for the different drugs used (34). This empirical evidence is likely multifactorial, as sex may influence effectiveness through the effects of estrogen on immune function, differences in drug pharmacokinetics/pharmacodynamics, and outcome measures. In our population, only 12 patients were of childbearing age; therefore, we did not analyze the differences in this particular reproductive age. Many studies suggest that a gender imbalance occurs only in subjective measures such as pain, functional status, and quality of life (31). Therefore, caution should be taken when a comparison is performed between male and female subjects since the fulfillment of the criteria to reach remission relies on patient-reported outcomes that may differ according to gender (35).

The long-term use of OGC is very common in patients with inflammatory arthritis, especially in RA patients, as well as in those treated with JAK-i. It is proven that chronic use of OGC has detrimental bone, metabolic, cardiovascular, and infectious side effects (36, 37). We therefore demonstrated, in conformity with other real-life settings and studies and regardless of the line of treatment, the steroid-sparing effect of JAK-i in these patients who undergo long-term OGC therapies (24, 38, 39). It is interesting to note, regarding the use of steroids, that patients in monotherapy with JAK-i had a higher baseline steroid dose compared with other patients. This finding can also be deduced from the fact that patients with RA receiving combination therapy with csDMARDs start with a lower steroid dosage and maintain it during the follow-up period. The steroid withdrawal rate was higher for all subgroups in the first 3 months of therapy, and in these first weeks, we also registered a significant improvement in patient-reported outcomes, including VAS-pain and General Health scores, and this effect might be due to JAK-i action on inflammation and associated chronic pain and fatigue (40, 41). We registered a higher prevalence of non-smokers in patients who suspended steroids during the follow-up. Cigarette smoking is a well-known risk factor for the pathogenesis of RA and is implicated in its development and severity. In patients with PsA, it may cause poor response and reduced adherence to treatment (42, 43). Interventions to stop smoking and correct other modifiable risk factors at a population level would be beneficial for patients. On the contrary, in the study population, steroid withdrawal did not differ according to sex, BMI, disease duration, number of comorbidities, line of therapy, and use of JAK-i in monotherapy or in combination with csDMARDs.

In the cohort, there were no differences in drug survival between male and female patients, and according to the line of treatment, in our opinion, this warrants further analyses with bigger study samples. The most common cause of JAK-i discontinuation was therapeutic inefficacy, followed by viral infections, especially recurrent reactivation of HSV. Most of the patients who suspended therapy for viral infections were being treated with concomitant OGC, with an average daily dose higher than the whole suspension cohort. Only one case of MACE was observed in the study cohort in an elderly patient with a history of hypertension, dyslipidemia, prior transient ischemic attack, and heterozygous MTHFR A1298C mutation. The patient who experienced pulmonary embolism had a seropositive RA and was treated with baricitinib in combination with leflunomide and 7.5 mg/day of prednisone. We acknowledge that the alert regarding the risk of thromboembolic events with JAK-i was adopted in Italy in November 2022. Patients included in this study were relatively old, with several associated comorbidities and long average disease duration, and most of them had already failed previous bDMARDs therapy (bDMARD-IR, D2T). The latter group of patients presented worse baseline clinimetrics than b-naive patients.

There are some limitations of this study associated with the type of prospective cohort, i.e., the small number of patients with PsA enrolled due to the fact that JAK-i received approval and reimbursement in Italy only in March 2022, the short follow-up time that needs to be extended, participant loss during follow-up leading to a decrease in sample size and potentially influencing results, control of external variables that might affect the results over time and, finally, potential selection bias that could render the results non-generalizable. Certainly, other types of studies, such as randomized controlled trials, are more suitable for establishing cause-and-effect relationships.

Despite these limitations, the study has numerous advantages, such as the possibility to have data over time allowing the establishment of the temporal sequence of events, the accurate real-time data collection for the assessment of remission and LDA rates, and the estimation of the factors associated with clinical remission. This real-life study closely reflects the conditions and variables of everyday clinical practice, making it more generalizable to the external world. Furthermore, it was possible to include different populations, such as D2T patients, who are typically excluded from randomized controlled trials, as well as those with comorbidities that reflect the complexities of daily clinical practice. This study provides important real-life information on the possibility of reducing steroids in treated patients. These data allow us to consider this type of treatment for patients where steroids are more harmful, thereby enabling a reduced use of steroids.

## Data availability statement

The raw data supporting the conclusions of this article will be made available by the authors, without undue reservation.

## Ethics statement

The studies involving humans were approved by the ethic committee of the University of Rome Tor Vergata. The studies were conducted in accordance with the local legislation and institutional requirements. The participants provided their written informed consent to participate in this study.

## Author contributions

PC: Conceptualization, Data curation, Formal analysis, Funding acquisition, Investigation, Methodology, Supervision, Validation, Visualization, Writing – original draft, Writing – review & editing. CM: Data curation, Formal analysis, Investigation, Methodology, Writing – original draft, Writing – review & editing. AV: Data curation, Writing – original draft, Writing – review & editing. LF: Data curation, Writing – original draft, Writing – review & editing. PT: Writing – original draft, Writing – review & editing, Data curation. BK: Investigation, Writing – original draft, Writing – review & editing. EG: Investigation, Writing – original draft, Writing – review & editing. AB: Conceptualization, Investigation, Supervision, Validation, Visualization, Writing – original draft, Writing – review & editing. MC: Conceptualization, Data curation, Investigation, Supervision, Validation, Visualization, Writing – original draft, Writing – review & editing.
