# Supplementary material for: The steroid-sparing effect of JAK inhibitors across multiple patient populations
Source: Front Immunol. 2024 Apr 12;15:1376476. doi: 10.3389/fimmu.2024.1376476 (PMC11045928; doi:10.3389/fimmu.2024.1376476)
Supplement: Supplementary file 1 [file Table_1.docx]

**Supplementary table 1.** Disease activity in Rheumatoid Arthritis (a) and Psoriatic Arthritis (b) patients receiving JAK-inhibitors during the follow-up

a)

| **Rheumatoid arthritis (n=88)** | | | | |
| --- | --- | --- | --- | --- |
|  | **Baseline (n=88)** | **T3 (n=88)** | **T6 (n=77)** | **T12 (n=54)** |
| **DAS28-CRP** | 4.87 ± 1 | 3.7 ± 1.1 *** | 3.4 ± 0.9*** | 3.2 ± 0.9 *** |
| **VAS-pain** | 67.9 ± 16.3 | 46.5 ± 19.7*** | 38.5 ± 18.9 | 33.9 ± 17.3*** |
| **GH** | 66.4 ± 15.5 | 44.0 ± 17.3*** | 40.2 ± 19.8*** | 37.7 ± 16.0*** |

b)

| **Psoriatic arthritis (n=15)** | | | | |
| --- | --- | --- | --- | --- |
|  | **Baseline (n=15)** | **T3 (n=15)** | **T6 (n=7)** | **T12 (n=6)** |
| **DAPSA-CRP** | 41.0 ± 22.2 | 37.6 ± 22.2* | 32.7 ± 15.1* | 27.24 ± 3.5* |
| **VAS-pain** | 66.1 ± 14.4 | 55.7 ±17.2** | 42.5 ± 33.0* | 40.2 ± 14.1 |
| **GH** | 66.1 ± 17.1 | 65.7 ± 9.7 | 45.0 ± 30.0 | 42.3 ± 14.1 |

^1^ DAS28-CRP: Disease Activity Score in 28 joints with CRP; DAPSA-CRP: Disease Activity in Psoriatic Arthritis with CRP; VAS-pain: Visual Analogic Scale; GH: patient General Health score. T3: 3 months, T6: 6 months, T12: 12 months.

^2^ Continuous variables are expressed as mean ± standard deviation.

^3^ *p<0.05, **p<0.01, ***p<0.001 vs baseline (Wilcoxon signed-rank test).

**Supplementary table 2.** Oral glucocorticoid daily dose at all time points in the study population and in Rheumatoid arthritis and Psoriatic arthritis patients

|  | **OGC dose (mg/day)** | | | |
| --- | --- | --- | --- | --- |
|  | **T0** | **T3** | **T6** | **T12** |
| **Rheumatoid arthritis** | 4.1 ± 5.2 | 2.2 ± 4.2*** | 2.6 ± 4.4** | 1.8 ± 3.1** |
| **Psoriatic arthritis** | 5.2 ± 6.7 | 1.8 ± 4.7* | 1.25 ± 2.5 | 5.0 ± 7.0 |

^1^ Oral glucocorticoid daily dose is expressed as mean prednisone-equivalent dose in milligrams (PDN dose) ± standard deviation.

^2^ T3: 3 months, T6: 6 months, T12: 12 months.

^3^ *p<0.05, **p<0.01, ***p<0.001 vs baseline (Wilcoxon signed-rank test).
